# Long-term outcomes of arthroscopic management of femoroacetabular impingement syndrome: a systematic review

**DOI:** 10.1007/s00402-025-05890-0

**Published:** 2025-04-24

**Authors:** Filippo Migliorini, Raju Vaishya, Francesco Simeone, Michael Kurt Memminger, Marcel Betsch, Mario Pasurka

**Affiliations:** 1 Department of Trauma and Reconstructive Surgery, University Hospital in Halle, Martin-Luther University Halle-Wittenberg, Halle, Germany; 2 Department of Life Sciences, Health, and Health Professions, Link Campus University, Rome, Italy; 3 Department of Orthopaedic and Trauma Surgery, Academic Hospital of Bolzano (SABES-ASDAA), Bolzano, Italy; 4 Department of Orthopaedics and Joint Replacement Surgery, Indraprastha Apollo Hospitals, New Delhi, India; 5 Department of Orthopaedics and Trauma Surgery, Universitätsklinikum Erlangen, Erlangen, Germany

**Keywords:** Femoroacetabular impingement, FAI syndrome, Arthroscopy, Hip, Arthroplasty

## Abstract

**Introduction:**

Femoroacetabular impingement (FAI) syndrome is a condition characterised by irregularities in the femur or acetabular rim, leading to hip pain, increased risk of osteoarthritis (OA), and potential need for total hip arthroplasty (THA). Non-surgical treatments are the first-line approach. However, arthroscopic surgery has become more prevalent due to its promising short- and medium-term outcomes. Recent meta-analyses suggest that hip arthroscopy may offer superior results compared to non-operative treatments, though follow-up periods in these studies have been limited to 12 months. This systematic review aims to evaluate the long-term effectiveness of arthroscopic management for FAI syndrome, hypothesising that it will significantly improve patient-reported outcomes (PROMs) over a follow-up period exceeding ten years.

**Methods:**

The review focused on studies published in peer-reviewed journals with a minimum follow-up of 120 months and assessed outcomes such as PROMs and complication rates. It adhered to PRISMA guidelines and used the PICOT algorithm to evaluate the literature. Data extraction covered study characteristics, PROMs, and complications. Statistical analyses were conducted using IBM SPSS software to summarise continuous and dichotomous data.

**Results:**

Of 1,245 identified articles, 7 were included after rigorous screening. Risk of bias assessment with the ROBINS-I tool revealed a serious or moderate risk of bias due to confounding, although overall methodological quality was acceptable. Data from 478 patients showed significant improvements in PROMs from baseline to follow-up.

**Conclusion:**

This systematic review indicates that arthroscopic management for FAI syndrome significantly improves PROMs with a mean follow-up of approximately 130 months. Nevertheless, 32% of patients required THA within ten years, underscoring the importance of careful patient selection and consideration of factors like OA and age. While conservative treatments such as physical therapy may yield comparable short-term outcomes, recent evidence suggests that arthroscopy provides superior results, particularly for younger patients and those without preoperative OA.

**Level of evidence:**

Level II, systematic review and meta-analysis.

**Supplementary Information:**

The online version contains supplementary material available at 10.1007/s00402-025-05890-0.

## Introduction

Femoroacetabular impingement (FAI) syndrome is defined as a syndrome characterised by irregularities of the femur (cam impingement) and/or the acetabular rim (pincer impingement), which lead to hip pain due to labral and chondral pathologies [1–3]. In 2016, the Warwick Agreement consensus statement described FAI syndrome as “a motion-related clinical diagnosis of the hip that represents symptomatic contact between the proximal femur and the acetabulum” [4]. The improper contact between the femur and acetabulum leads to continuous joint damage and soft tissue injuries [5–7]. Besides associated hip pain and decreased quality of life, FAI syndrome also seems to increase the risk of developing osteoarthritis (OA) and the need for total hip arthroplasty (THA) [8–11].

There are several treatment strategies for FAI syndrome, including non-surgical options (e.g., physical therapy, activity modifications, injection therapy) and surgery [1]. Physical therapy and activity modification are considered the first-line treatments for FAI syndrome. When conservative management fails, arthroscopic surgery for FAI syndrome has become increasingly popular, showing promising clinical results in addressing both bony and soft tissue pathologies [12–16]. Hip arthroscopy for managing FAI syndrome may lead to superior clinical outcomes than non-operative treatment [17, 18]. Hip arthroscopy for FAI syndrome demonstrated significant improvement at a 5-year follow-up, with maintained rates of achieving minimal clinically important difference (MCID), Patient Acceptable Symptom State (PASS), and Substantial Clinical Benefit (SCB) [19]. Despite these initial studies with short- and midterm outcomes, there is limited evidence regarding the long-term outcomes of arthroscopic management for treating FAI syndrome. Therefore, this systematic review evaluated the outcomes of arthroscopic management for FAI syndrome with a follow-up period of over ten years. The authors hypothesised that arthroscopic management for FAI syndrome would result in a statistically significant improvement in PROMs (Patient-Reported Outcome Measures) at long-term follow-up.

## Methods

### Eligibility criteria

All clinical investigations which evaluated the long-term outcome of arthroscopic management of FAI syndrome were considered. Only studies published in peer-reviewed journals were deemed eligible. Studies with levels I to III of evidence, according to the 2020 Oxford Centre of Evidence-Based Medicine [20], were included. Editorials, reviews, letters, opinions, and studies involving in vitro or animal experiments, biomechanical assessments, computational analyses, or cadaveric research were excluded. Studies which evaluated the results of open or mini-open surgery were considered. Only studies with a minimum of 120 months of follow-up were included in the present review.

### Search strategy

The current systematic review adhered to the guidelines outlined in the Preferred Reporting Items for Systematic Reviews and Meta-Analyses (PRISMA) statement of 2020 [21]. The PICOT algorithm was followed:

- P(Problem): FAI syndrome;
- I(Intervention): arthroscopic management;
- C(Comparison): none;
- O(Outcomes): PROMs, rate of complication;
- T(Timing): minimum 10 years follow-up.

In August 2024, the following databases were accessed: PubMed, Embase, and Web of Science, with no additional filters or time constraints. The Medical Subject Headings (MeSH) used for the database search are outlined in the Appendix.

### Selection and data collection

Two authors (F.M. and T.B.) performed the database search. All retrieved titles underwent manual screening, and their abstracts were accessed if deemed appropriate. Full texts were examined in cases where there was a match. Articles without accessible full texts were excluded from consideration. A cross-reference of the bibliographies of full-text articles was also conducted for potential inclusion. A third senior author (R.V.), who made the final decision, resolved disagreements among authors.

### Data items

Two authors (F.M. and T.B.) performed data extraction. The following data at baseline were extracted: author, year of publication and journal, length of the follow-up, number of patients with related mean age, and body mass index (BMI). Data concerning the following PROMs were collected at baseline and at the last follow-up: visual analogue scale (VAS) [22], modified Harris Hip Score (mHHS) [23], Hip Outcome Score - Activities of Daily Living (HOS-ADL) [24], and modified Hip Outcome Score - Sport-Specific Subscale (HOS-SSS) [25]. Data concerning the following complications were retrieved: re-operations, revision arthroscopy, and progression to THA. Data were extracted in Microsoft Office Excel version 16.0 (Microsoft Corporation, Redmond, USA).

### Assessment of the risk of bias

The guidelines of the Cochrane Handbook for Systematic Reviews of Interventions [26] were followed to assess the Risk of Bias. Two authors (F.M. and T.B.) independently evaluated the risk of bias in the extracted studies. The Risk of Bias in Nonrandomised Studies of Interventions (ROBINS-I) tool [27] was used since only Nonrandomised controlled trials (non-RCTs) were included in this review. Seven domains of potential bias in non-RCTs were assessed. Tow domains assess the possible confounding and the nature of patient selection before the start of the comparative intervention. Bias in the classification during the intervention is assessed by a further domain. The final four domains are used to assess the methodological quality after the intervention comparison has been implemented and relate to deviations from previously intended interventions, missing data, erroneous measurement of outcomes, and bias in the selection of reported outcomes. The chart of the ROBINS-I was elaborated using the Robvis Software (Risk-of-bias VISualization, Riskofbias.info, Bristol, UK) [28].

### Synthesis method

The main author (F.M.) performed the statistical analyses following the recommendations of the Cochrane Handbook for Systematic Reviews of Interventions [26]. For descriptive statistics, the IBM SPSS software version 25 was used. The arithmetic mean and standard deviation were used for continuous data, and the frequency (events/ observations) for dichotomic variables.

## Results

### Study selection

**Fig. 1:**
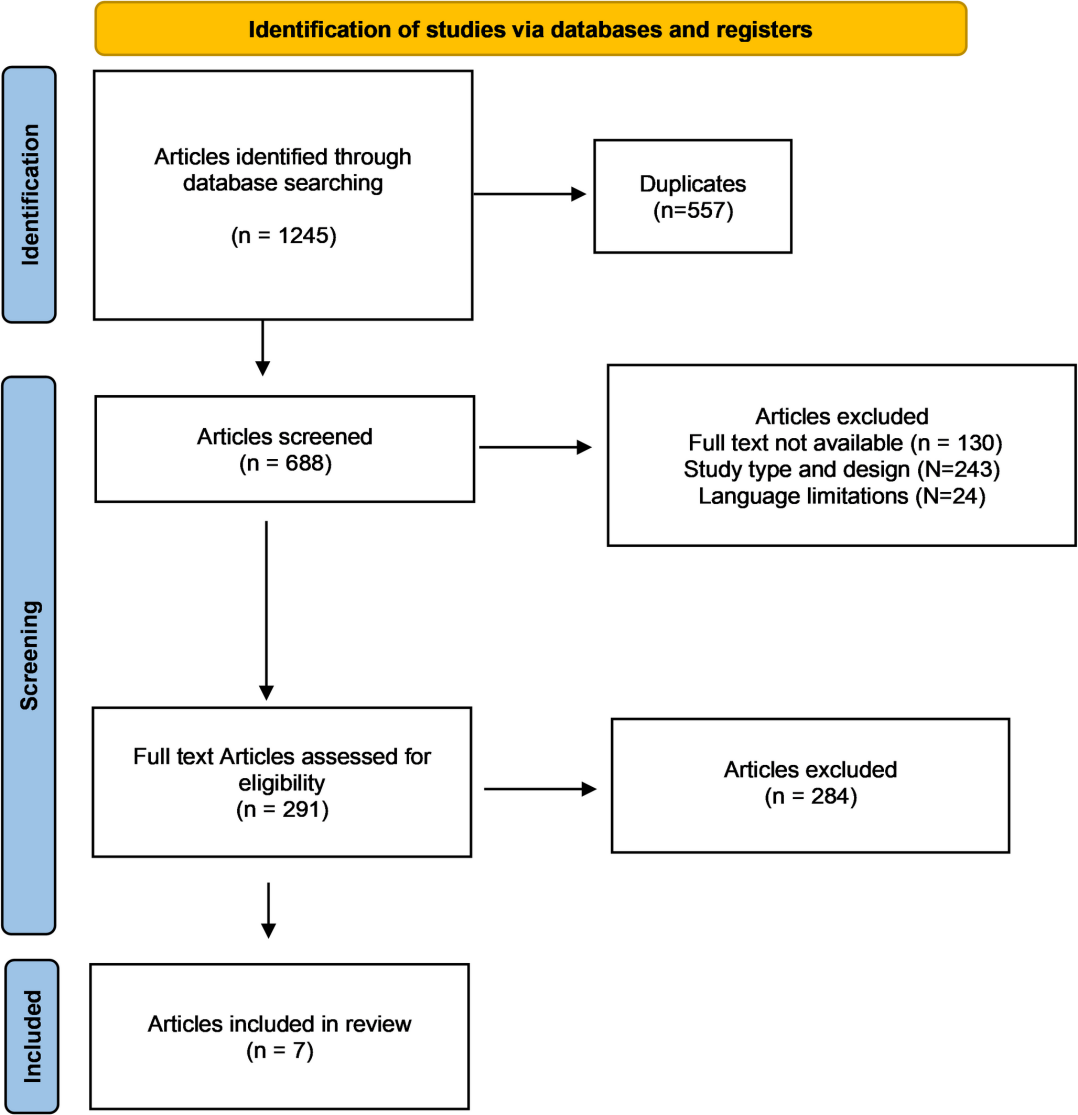
PRISMA flow chart of the literature search

The initial stage of this systematic review involved a comprehensive literature search that identified 1245 articles potentially relevant to the research topic. Following deduplication efforts, 688 articles were selected for eligibility screening based on their abstracts. A total of 397 articles were subsequently excluded for various reasons, the primary reason being a lack of alignment with the predefined study design criteria (*N* = 243). Language barriers (*N* = 24) and limitations in accessing the full text (*N* = 130) further contributed to article exclusions. A meticulous full-text review was conducted on the remaining 291 articles, excluding 284 articles. Consequently, the final selection for this systematic review comprised seven studies. The results of the literature search are shown in Fig. 1.

### Risk of bias assessment

**Fig. 2:**
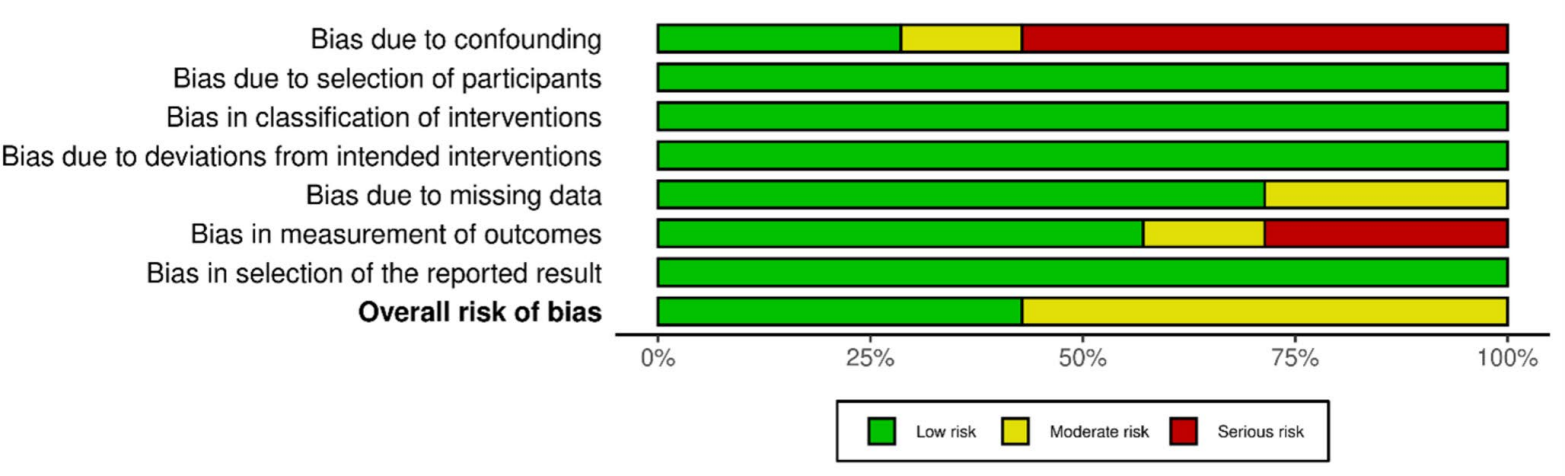
The ROBINS-I of non-RCTs

The risk of bias of non-RCTs was assessed using the ROBINS-I risk of bias tool on all the included trials since no RCT was selected. The risk of bias due to confounding was serious or moderate for more than half of the included studies. The risk of bias in participant selection, intervention classification, and deviations from intended intervention was low in all the included studies. In domains assessed for risk of bias after the intervention, some concerns were identified in the measurement of outcomes. No concerns were raised about the selection of the reported results. The overall risk of bias was moderate in 60% and low in 30% of the included studies, indicating a mostly acceptable methodological quality (Fig. 2).

### Study characteristics and results of individual studies

Data from 478 patients were retrieved. Of them, 44.4% (212 of 478 patients) were women. The mean length of follow-up was 132.1 ± 3.4 months. The mean age was 36.7 ± 9.0 years, and the mean BMI was 22.8 ± 1.1 kg/m^2^. Generalities of the included studies are shown in Table 1.

**Table 1:** Generalities of the included studies (BMI: body mass index)

Author and Year	Journal	Design	Follow-up (months)	Patients (*n*)	Women (*n*)	Mean Age (years)	Mean BMI (kg/m^2^)
Büchler et al., 2021 [29]	*Clin Orthop Relat Res*	Retrospective	132.0	50	45	33.0	21.9
Byrd et al., 2009 [30]	*Arthroscopy*	Prospective	120.0	26	13	46.0	
Lee et al., 2021 [31]	*J Hip Preserv Surg*	Retrospective	131.5	28	9	36.5	22.7
			135.0	87	27	34.0	22.8
Martinez et al., 2023 [32]	*Rev Esp Cir Ortop Traumatol*	Retrospective	132.0	17	2	47.8	25.1
				54	9	40.6	25.3
Menge et al., 2021 [33]	*Am J Sports Med*	Prospective	134.0	60	49	16.0	22.0
Zimmerer et al., 2021 [34]	*Arthroscopy*	Retrospective	132.7	51	21	43.0	22.0
			131.2	61	20	44.1	22.4
Zimmerer et al., 2021 [35]	*Orthop J Sports Med*	Prospective	132.0	44	17	42.2	22.3

### Results syntheses

All PROMs of interest improved significantly compared to the baseline (Table 2): VAS (*P* = 0.0002), mHHS (*P* = 0.0003), HOS-ADL (*P* = 0.001), and HOS-SSS (*P* = 0.03).

**Table 2:** Result of proms (VAS: visual analogue scale; mHHS: modified Harris hip score; HOS-ADL: hip outcome Score - Activities of daily living; HOS-SSS: hip outcome Score - Sport-Specific subscale; FU: follow-up; MD: mean difference)

Endpoint	At baseline	At last FU	MD	*P*
VAS (0–10)	6.7 ± 0.3	2.4 ± 0.5	-4.3	0.0002
mHHS (0-100)	62.4 ± 9.1	86.9 ± 4.6	24.5	0.0003
HOS-ADL (0-100)	66.0 ± 5.4	85.8 ± 2.5	19.8	0.001
HOS-SSS (0-100)	51.2 ± 14.7	77.5 ± 3.3	26.3	0.03

At the last follow-up, 29% (77 of 265) of patients underwent reoperation, 10% (18 of 189) revision arthroscopy, and 32% (65 of 205) progressed to THA. Not all studies provide quantitative data for each endpoint; consequently, the sample size varies across different results.

## Discussion

According to the main findings of this systematic review, arthroscopic management for FAI syndrome results in a statistically significant improvement in PROMs at a mean follow-up of approximately 130 months. Within the 10-year follow-up period, 29% of patients underwent reoperation, 10% had revision arthroscopy, and 32% progressed to THA.

In addition to surgical treatment for FAI syndrome, conservative options such as physical therapy, activity modifications, and injection therapy should also be considered. Some studies have reported no significant difference in the effectiveness between surgery and physical therapy for FAI syndrome [36, 37].

In line with these results, a meta-analysis by Bastos et al. demonstrated moderate-quality evidence that surgery is not superior to conservative treatment for FAI syndrome in the short term, with low-quality evidence in the medium term [38]. However, there has been a rapid increase in the number of surgeries performed [12, 13], and more recent evidence has reported statistically superior clinical outcomes for hip arthroscopy compared to conservative treatment [18]. However, the inconsistent results may also be affected by the characteristics of the patients included in the studies.

Several risk factors have been identified as predictors of poor clinical outcomes in FAI syndrome. Advanced chondral damage, particularly in the presence of extensive chondrolabral injury, has been associated with suboptimal prognoses. Pre-existing osteoarthritis and abnormal femoral head morphology, including elevated alpha angles and severe cam or pincer deformities, may contribute to the persistence of symptoms despite treatment. Other prognostic factors include elevated body mass index (BMI), reduced preoperative range of motion, and prolonged symptom duration before intervention have also been correlated with lower postoperative outcomes [39–42]. Several studies have investigated the difference in functional improvement after arthroscopic treatment for FAI syndrome across different age groups and have found superior results for younger patients [43–48]. However, the present systematic review demonstrated statistically significant improvement in PROMs in age 16 to 47.8 years. Additionally, some studies have shown no differences among various age groups [48–50], and even patients older than 60 can benefit from an arthroscopic treatment, with outcomes comparable to those of younger adults (18–59 years) [47]. However, despite improvements in clinical outcomes, the present systematic review also demonstrated that 32% of patients progressed to THA within the 10-year follow-up. Therefore, pursuing arthroscopic treatment for FAI syndrome should be based on a shared decision-making process between physicians and patients, considering several clinical factors, especially preoperative OA. Among the studies in the present systematic review, increased age and greater joint degeneration were the most commonly cited predictors of clinical failure. Good clinical outcomes and a low revision surgery rate have been reported in adolescents [33]. A study from Buechler et al. also reported a lower rate of progression to THA for younger patients (hazard ratio 1.1, *p* = 0.01) and hips without signs of osteoarthritis (preoperative Tönnis Grade 1 compared with Tönnis Grade 0 (hazard ratio 17; *p* = 0.01) [29]. However, even in this cohort, the cumulative 10-year survival rate was 92% (median age 33, range 16–63) [29]. Analysing the median Harris Hip Score improvement, Bryd and Jones found worse outcomes for patients with associated arthritis. In a prospective analysis with a 10-year follow-up after hip arthroscopy, 88% of patients with preoperative arthritis were converted to total hip arthroplasty at a mean of 63 months [30]. In contrast, 83% of patients without OA showed substantial improvement in Harris Hip Scores at 10-year follow-up [30]. In line with these findings, a recent study from Más Martínez et al. reported a cumulative survivorship rate of 77.8% at ten years, with a rate of 45.4% for patients with a Toennis grade greater than 1 and 85.2% for patients with a Toennis grade of 1 or less (*p* < 0.001) [32]. Besides the presence of OA, advanced age and female sex also seem to affect the outcome after hip arthroscopy for FAI syndrome adversely [34]. Zimmer et al. reported a 97% higher risk of THA conversion for female patients [34]. Interestingly, this risk was only 24% higher for patients with advanced age at the time of surgery but 133% higher for hips with a Toennis grade greater than 1 [34]. There is a significant variation in gender distribution among studies analysing clinical outcomes after arthroscopic treatment for FAI syndrome. In the present systematic review, 44% of the 478 patients were women, although there was a high variability between the included studies. Additionally, the preoperative duration of symptoms seems to be an independent predictor of achieving meaningful clinical outcomes from arthroscopic treatment of FAI syndrome. However, significant improvement at the 5-year follow-up has been reported recently, with maintained rates of achieving MCID, PASS, and SCB. The survival rate of hip arthroscopy at five years is generally high, with conversion rates to THA or revision surgery ranging from 0.0 to 17.9% and from 1.3 to 26.7%, respectively.

This study has several limitations. Pre-existing OA as a factor influencing clinical outcomes after surgery was not considered, as this review focused solely on evaluating long-term clinical outcomes. Additionally, the number of included studies is small, particularly for prospective studies, which may contribute to high heterogeneity between studies. The authors did not prospectively register the present systematic review into a public registry, potentially increasing the risk of bias. The data did not account for different morphology types of FAI syndrome, potentially introducing reporting bias. Larger, multicenter, high-quality RCTs are needed to validate the outcomes of this systematic review.

## Conclusion

This systematic review indicates that arthroscopic management for FAI syndrome significantly improves PROMs with a mean follow-up of approximately 130 months. Nevertheless, 32% of patients required THA within ten years, underscoring the importance of careful patient selection and consideration of factors like OA and age. While conservative treatments such as physical therapy may yield comparable short-term outcomes, recent evidence suggests that arthroscopy provides superior results, particularly for younger patients and those without preoperative OA.

## Electronic supplementary material

Below is the link to the electronic supplementary material.

Supplementary Material 1

## Data Availability

The datasets generated during and/or analysed during the current study are available throughout the manuscript.

## References

[1] Trigg SD, Schroeder JD, Hulsopple C (2020) Femoroacetabular impingement syndrome. Curr Sports Med Rep 19(9):360–366. 10.1249/jsr.0000000000000748 32925375 10.1249/JSR.0000000000000748

[2] Ganz R, Parvizi J, Beck M, Leunig M, Nötzli H, Siebenrock KA (2003) Femoroacetabular impingement: a cause for osteoarthritis of the hip. Clin Orthop Relat Res 417:112–120. 10.1097/01.blo.0000096804.78689.c2 10.1097/01.blo.0000096804.78689.c2 14646708

[3] Kemp JL, Risberg MA (2018) Significant knowledge gaps between clinical practice and research on femoroacetabular impingement: are we on the same path?? J Orthop Sports Phys Ther 48(4):228–229. 10.2519/jospt.2018.0103 29607765 10.2519/jospt.2018.0103

[4] Griffin DR, Dickenson EJ, O’Donnell J, Agricola R, Awan T, Beck M, Clohisy JC, Dijkstra HP, Falvey E, Gimpel M, Hinman RS, Hölmich P, Kassarjian A, Martin HD, Martin R, Mather RC, Philippon MJ, Reiman MP, Takla A, Thorborg K, Walker S, Weir A, Bennell KL (2016) The Warwick agreement on femoroacetabular impingement syndrome (FAI syndrome): an international consensus statement. Br J Sports Med 50(19):1169–1176. 10.1136/bjsports-2016-096743 27629403 10.1136/bjsports-2016-096743

[5] Sankar WN, Nevitt M, Parvizi J, Felson DT, Agricola R, Leunig M (2013) Femoroacetabular impingement: defining the condition and its role in the pathophysiology of osteoarthritis. J Am Acad Orthop Surg 21(Suppl 1):S7–s15. 10.5435/jaaos-21-07-s7 23818194 10.5435/JAAOS-21-07-S7

[6] Migliorini F, Maffulli N, Bell A, Cuozzo F, Hildebrand F, Weber CD (2023) Midterm results after arthroscopic femoral neck osteoplasty combined with labral debridement for cam type femoroacetabular impingement in active adults. J Orthop Surg Res 18(1):67. 10.1186/s13018-023-03543-9 36707868 10.1186/s13018-023-03543-9 PMC9880366

[7] Migliorini F, Maffulli N, Baroncini A, Eschweiler J, Tingart M, Betsch M (2022) Revision surgery and progression to total hip arthroplasty after surgical correction of femoroacetabular impingement: A systematic review. Am J Sports Med 50(4):1146–1156. 10.1177/03635465211011744 34081552 10.1177/03635465211011744 PMC8980457

[8] Migliorini F, Liu Y, Eschweiler J, Baroncini A, Tingart M, Maffulli N (2022) Increased range of motion but otherwise similar clinical outcome of arthroscopy over open osteoplasty for femoroacetabular impingement at midterm follow-up: A systematic review. Surgeon 20(3):194–208. 10.1016/j.surge.2021.01.016 33731304 10.1016/j.surge.2021.01.016

[9] Migliorini F, Liu Y, Catalano G, Trivellas A, Eschweiler J, Tingart M, Maffulli N (2021) Medium-term results of arthroscopic treatment for femoroacetabular impingement. Br Med Bull 138(1):68–84. 10.1093/bmb/ldaa038 33454746 10.1093/bmb/ldaa038

[10] Migliorini F, Baroncini A, Eschweiler J, Knobe M, Tingart M, Maffulli N (2023) Return to sport after arthroscopic surgery for femoroacetabular impingement. Surgeon 21(1):21–30. 10.1016/j.surge.2021.11.006 34953722 10.1016/j.surge.2021.11.006

[11] Lucenti L, Maffulli N, Bardazzi T, Saggini R, Memminger M, Simeone F, Migliorini F (2024) Return to sport following arthroscopic management of femoroacetabular impingement: A systematic review. J Clin Med 13(17). 10.3390/jcm13175219 10.3390/jcm13175219 PMC11395971 39274432

[12] Bozic KJ, Chan V, Valone FH 3rd, Feeley BT, Vail TP (2013) Trends in hip arthroscopy utilization in the united States. J Arthroplasty 28(8 Suppl):140–143. 10.1016/j.arth.2013.02.039 10.1016/j.arth.2013.02.039 23916639

[13] Montgomery SR, Ngo SS, Hobson T, Nguyen S, Alluri R, Wang JC, Hame SL (2013) Trends and demographics in hip arthroscopy in the united States. Arthroscopy 29(4):661–665. 10.1016/j.arthro.2012.11.005 23375668 10.1016/j.arthro.2012.11.005

[14] Migliorini F, Maffulli N, Knobe M, Eschweiler J, Tingart M, Baroncini A (2022) Arthroscopic labral repair for femoroacetabular impingement: A systematic review. Surgeon 20(5):e225–e230. 10.1016/j.surge.2021.02.013 33820729 10.1016/j.surge.2021.02.013

[15] Migliorini F, Maffulli N (2021) Arthroscopic management of femoroacetabular impingement in adolescents: A systematic review. Am J Sports Med 49(13):3708–3715. 10.1177/0363546521997138 33740385 10.1177/0363546521997138

[16] Wagner M, Lindtner RA, Schaller L, Schmaranzer F, Schmaranzer E, Vavron P, Endstrasser F, Brunner A (2024) Hip arthroscopy with initial access to the peripheral compartment for femoroacetabular impingement: midterm results from a large-scale patient cohort. J Orthop Traumatol 25(1):29. 10.1186/s10195-024-00770-6 38789896 10.1186/s10195-024-00770-6 PMC11126547

[17] Gatz M, Driessen A, Eschweiler J, Tingart M, Migliorini F (2020) Arthroscopic surgery versus physiotherapy for femoroacetabular impingement: a meta-analysis study. Eur J Orthop Surg Traumatol 30(7):1151–1162. 10.1007/s00590-020-02675-6 32382825 10.1007/s00590-020-02675-6 PMC7505824

[18] Zhu Y, Su P, Xu T, Zhang L, Fu W (2022) Conservative therapy versus arthroscopic surgery of femoroacetabular impingement syndrome (FAI): a systematic review and meta-analysis. J Orthop Surg Res 17(1):296. 10.1186/s13018-022-03187-1 35659016 10.1186/s13018-022-03187-1 PMC9166461

[19] Jan K, Fenn TW, Kaplan DJ, Nho SJ (2023) Patients maintain clinically significant outcomes at 5-Year Follow-Up after hip arthroscopy for femoroacetabular impingement syndrome: A systematic review. Arthroscopy 39(8):1869–1881e1861. 10.1016/j.arthro.2023.04.021 37207920 10.1016/j.arthro.2023.04.021

[20] Howick JCI, Glasziou P, Greenhalgh T, Carl Heneghan, Liberati A, Moschetti I, Phillips B, Thornton H, Goddard O, Hodgkinson M (2011) The 2011 Oxford CEBM Levels of Evidence. Oxford Centre for Evidence-Based Medicine Available at https://wwwcebmnet/indexaspx?o=5653

[21] Page MJ, McKenzie JE, Bossuyt PM, Boutron I, Hoffmann TC, Mulrow CD, Shamseer L, Tetzlaff JM, Akl EA, Brennan SE, Chou R, Glanville J, Grimshaw JM, Hróbjartsson A, Lalu MM, Li T, Loder EW, Mayo-Wilson E, McDonald S, McGuinness LA, Stewart LA, Thomas J, Tricco AC, Welch VA, Whiting P, Moher D (2021) The PRISMA 2020 statement: an updated guideline for reporting systematic reviews. BMJ 372:n71. 10.1136/bmj.n71 33782057 10.1136/bmj.n71 PMC8005924

[22] Migliorini F, Maffulli N, Eschweiler J, Schenker H, Tingart M, Betsch M (2023) Arthroscopic versus mini-open rotator cuff repair: A meta-analysis. Surgeon 21(1):e1–e12. 10.1016/j.surge.2021.11.005 34961701 10.1016/j.surge.2021.11.005

[23] Hung M, Hon SD, Cheng C, Franklin JD, Aoki SK, Anderson MB, Kapron AL, Peters CL, Pelt CE (2014) Psychometric evaluation of the lower extremity computerized adaptive test, the modified Harris hip score, and the hip outcome score. Orthop J Sports Med 2(12):2325967114562191. 10.1177/2325967114562191 26535291 10.1177/2325967114562191 PMC4555528

[24] Martin RL, Philippon MJ (2008) Evidence of reliability and responsiveness for the hip outcome score. Arthroscopy 24(6):676–682. 10.1016/j.arthro.2007.12.011 18514111 10.1016/j.arthro.2007.12.011

[25] Martin RL, Kelly BT, Philippon MJ (2006) Evidence of validity for the hip outcome score. Arthroscopy 22(12):1304–1311. 10.1016/j.arthro.2006.07.027 17157729 10.1016/j.arthro.2006.07.027

[26] Higgins JPTTJ, Chandler J, Cumpston M, Li T, Page MJ, Welch VA (eds) (2022) Cochrane Handbook for Systematic Reviews of Interventions version 6.3 (updated February 2022). Cochrane, Available from www.training.cochrane.org/handbook

[27] Sterne JA, Hernan MA, Reeves BC, Savovic J, Berkman ND, Viswanathan M, Henry D, Altman DG, Ansari MT, Boutron I, Carpenter JR, Chan AW, Churchill R, Deeks JJ, Hrobjartsson A, Kirkham J, Juni P, Loke YK, Pigott TD, Ramsay CR, Regidor D, Rothstein HR, Sandhu L, Santaguida PL, Schunemann HJ, Shea B, Shrier I, Tugwell P, Turner L, Valentine JC, Waddington H, Waters E, Wells GA, Whiting PF, Higgins JP (2016) ROBINS-I: a tool for assessing risk of bias in non-randomised studies of interventions. BMJ 355:i4919. 10.1136/bmj.i4919 27733354 10.1136/bmj.i4919 PMC5062054

[28] McGuinness LA, Higgins JPT (2020) Risk-of-bias visualization (robvis): an R package and Shiny web app for visualizing risk-of-bias assessments. 10.1002/jrsm.1411. Research Synthesis Methods n/a (n/a) 10.1002/jrsm.1411 32336025

[29] Büchler L, Grob V, Anwander H, Lerch TD, Haefeli PC (2021) Good outcome scores and low conversion rate to THA 10 years after hip arthroscopy for the treatment of femoroacetabular impingement. Clin Orthop Relat Res 479(10):2256–2264. 10.1097/corr.0000000000001778 33929975 10.1097/CORR.0000000000001778 PMC8445580

[30] Byrd JW, Jones KS (2009) Hip arthroscopy for labral pathology: prospective analysis with 10-year follow-up. Arthroscopy 25(4):365–368. 10.1016/j.arthro.2009.02.001 19341922 10.1016/j.arthro.2009.02.001

[31] Lee JK, Hwang DS, Kim SB, Kang C, Hwang JM, Lee GS, Park EJ (2021) The role and clinical relevance of the ligamentum Teres: long-term outcomes after hip arthroscopic surgery of cam-type femoroacetabular impingement. J Hip Preserv Surg 8(4):360–366. 10.1093/jhps/hnab080 35505805 10.1093/jhps/hnab080 PMC9052427

[32] Más Martínez J, Cuenca Copete A, Verdú Román C, Jiménez Arias D, Beneito Pastor D, Sanz-Reig J (2024) Hip arthroscopy for femoroacetabular impingement with 10-year minimum follow-up. Rev Esp Cir Ortop Traumatol 68(1):35–43. 10.1016/j.recot.2023.06.015 37995818 10.1016/j.recot.2023.11.012

[33] Menge TJ, Briggs KK, Rahl MD, Philippon MJ (2021) Hip arthroscopy for femoroacetabular impingement in adolescents: 10-Year Patient-Reported outcomes. Am J Sports Med 49(1):76–81. 10.1177/0363546520973977 33259224 10.1177/0363546520973977

[34] Zimmerer A, Ramoser A, Streit M, Janz V, Sobau C, Wassilew GI, Miehlke W (2021) Osteoarthrosis, advanced age, and female sex are risk factors for inferior outcomes after hip arthroscopy and labral debridement for femoroacetabular impingement syndrome: case series with minimum 10-Year Follow-Up. Arthroscopy 37(6):1822–1828e1821. 10.1016/j.arthro.2021.01.024 33515737 10.1016/j.arthro.2021.01.024

[35] Zimmerer A, Janz V, Sobau C, Wassilew GI, Miehlke W (2021) Defining the clinically meaningful outcomes for arthroscopic treatment of femoroacetabular impingement syndrome at minimum 10-Year Follow-up: the timing of surgery is crucial. Orthop J Sports Med 9(2):2325967120985140. 10.1177/2325967120985140 33718501 10.1177/2325967120985140 PMC7922622

[36] Realpe AX, Foster NE, Dickenson EJ, Jepson M, Griffin DR, Donovan JL (2021) Patient experiences of receiving arthroscopic surgery or personalised hip therapy for femoroacetabular impingement in the context of the UK fashion study: a qualitative study. Trials 22(1):211. 10.1186/s13063-021-05151-6 33726810 10.1186/s13063-021-05151-6 PMC7962311

[37] Mansell NS, Rhon DI, Meyer J, Slevin JM, Marchant BG (2018) Arthroscopic surgery or physical therapy for patients with femoroacetabular impingement syndrome: A randomized controlled trial with 2-Year Follow-up. Am J Sports Med 46(6):1306–1314. 10.1177/0363546517751912 29443538 10.1177/0363546517751912

[38] Bastos RM, de Carvalho Júnior JG, da Silva SAM, Campos SF, Rosa MV, de Moraes Prianti B (2021) Surgery is no more effective than Conservative treatment for femoroacetabular impingement syndrome: systematic review and meta-analysis of randomized controlled trials. Clin Rehabil 35(3):332–341. 10.1177/0269215520966694 33143438 10.1177/0269215520966694

[39] Stone AV, Beck EC, Malloy P, Chahla J, Nwachukwu BU, Neal WH, Nho SJ (2019) Preoperative predictors of achieving clinically significant athletic functional status after hip arthroscopy for femoroacetabular impingement at minimum 2-Year Follow-Up. Arthroscopy 35(11):3049–3056e3041. 10.1016/j.arthro.2019.05.022 31395395 10.1016/j.arthro.2019.05.022

[40] Menge TJ, Briggs KK, Dornan GJ, McNamara SC, Philippon MJ (2017) Survivorship and outcomes 10 years following hip arthroscopy for femoroacetabular impingement: labral debridement compared with labral repair. J Bone Joint Surg Am 99(12):997–1004. 10.2106/jbjs.16.01060 28632588 10.2106/JBJS.16.01060

[41] Minkara AA, Westermann RW, Rosneck J, Lynch TS (2019) Systematic review and Meta-analysis of outcomes after hip arthroscopy in femoroacetabular impingement. Am J Sports Med 47(2):488–500. 10.1177/0363546517749475 29373805 10.1177/0363546517749475

[42] Degen RM, Nawabi DH, Bedi A, Kelly BT (2017) Radiographic predictors of femoroacetabular impingement treatment outcomes. Knee Surg Sports Traumatol Arthrosc 25(1):36–44. 10.1007/s00167-015-3794-2 26387126 10.1007/s00167-015-3794-2

[43] Beck EC, Drager J, Nwachukwu BU, Jan K, Rasio J, Nho SJ (2021) Gender and Age-Specific differences observed in rates of achieving meaningful clinical outcomes 5-Years after hip arthroscopy for femoroacetabular impingement syndrome. Arthroscopy 37(8):2488–2496e2481. 10.1016/j.arthro.2021.02.033 33677021 10.1016/j.arthro.2021.02.033

[44] Bloom DA, Fried JW, Bi AS, Kaplan DJ, Chintalapudi N, Youm T (2020) Age-Associated pathology and functional outcomes after hip arthroscopy in female patients: analysis with 2-Year Follow-up. Am J Sports Med 48(13):3265–3271. 10.1177/0363546520957712 33026835 10.1177/0363546520957712

[45] Bryan AJ, Krych AJ, Pareek A, Reardon PJ, Berardelli R, Levy BA (2016) Are Short-term outcomes of hip arthroscopy in patients 55 years and older inferior to those in younger patients? Am J Sports Med 44(10):2526–2530. 10.1177/0363546516652114 27416992 10.1177/0363546516652114

[46] Byrd JW, Jones KS, Gwathmey FW (2016) Arthroscopic management of femoroacetabular impingement in adolescents. Arthroscopy 32(9):1800–1806. 10.1016/j.arthro.2016.02.019 27189871 10.1016/j.arthro.2016.02.019

[47] Byrd JWT, Jones KS (2019) Arthroscopic acetabular labral repair in patients over the age of 60 years: A matched Case-Control study. Arthroscopy 35(5):1406–1410. 10.1016/j.arthro.2018.11.015 31000389 10.1016/j.arthro.2018.11.015

[48] Domb BG, Linder D, Finley Z, Botser IB, Chen A, Williamson J, Gupta A (2015) Outcomes of hip arthroscopy in patients aged 50 years or older compared with a matched-pair control of patients aged 30 years or younger. Arthroscopy 31(2):231–238. 10.1016/j.arthro.2014.08.030 25442657 10.1016/j.arthro.2014.08.030

[49] Degen RM, Mayer SW, Fields KG, Coleman SH, Kelly BT, Nawabi DH (2017) Functional outcomes and cam recurrence after arthroscopic treatment of femoroacetabular impingement in adolescents. Arthroscopy 33(7):1361–1369. 10.1016/j.arthro.2017.01.044 28412058 10.1016/j.arthro.2017.01.044

[50] Aguilera-Bohórquez B, Brugiatti M, Coaquira R, Cardozo O, Cantor E (2020) Functional outcomes of arthroscopic treatment in femoroacetabular impingement in patients over 60 years old compared with patients aged 40 years or younger. Rev Bras Ortop (Sao Paulo) 55(6):715–721. 10.1055/s-0040-1708515 33364649 10.1055/s-0040-1708515 PMC7748922

