# Supplementary material for: Long-term outcomes of arthroscopic management of femoroacetabular impingement syndrome: a systematic review
Source: Arch Orthop Trauma Surg. 2025 Apr 24;145(1):267. doi: 10.1007/s00402-025-05890-0 (PMC12021968; doi:10.1007/s00402-025-05890-0)
Supplement: Supplementary file 1 — Supplementary Material 1 [file 402_2025_5890_MOESM1_ESM.docx]

**Research Question:**

**Femoracetabular Impingement Long Term Follow-up Studies**

**Concept 1: Condition**

**Keywords:**

Cam impingement

FAI

Femoroacetabular impingement

hip

Hip injury

labral tear

**Mesh:**

"Acetabulum / injuries"[Mesh]

"Athletic Injuries / physiopathology"[Mesh]

"Femoracetabular Impingement"[Mesh]

"Femoracetabular Impingement / etiology"[Mesh]

"Femoracetabular Impingement / physiopathology"[Mesh]

**Concept 2: Long Term Follow-up**

**Keywords:**

10-year follow-up

long-term follow-up

**Mesh:**

"Follow-Up Studies"[Mesh]

**Concept 3: Therapy**

**Keywords:**

Arthroscopy

hip arthroscopic surgery

Hip arthroscopy

labral repair

**Mesh:**

"Acetabulum / surgery"[Mesh]

"Arthroscopy"[Mesh]

"Arthroscopy / methods"[Mesh]

"Athletic Injuries / surgery"[Mesh]

"Debridement"[Mesh]

"Debridement / methods"[Mesh]

"Femoracetabular Impingement / surgery"[Mesh]

"Hip Injuries / surgery"[Mesh]

"Hip Joint / surgery"[Mesh]

"Joint Capsule / surgery"[Mesh]

"Joint Diseases / surgery"[Mesh]

**Concept 4: Interest of the outcome PROMS**

**Keywords:**

outcome

Treatment outcome

**Mesh:**

"Patient Reported Outcome Measures"[Mesh]

"Patient Satisfaction"[Mesh]

"Treatment Outcome"[Mesh]

**Searching Strategy**

"Acetabulum / injuries"[Mesh] OR "Athletic Injuries / physiopathology"[Mesh] OR "Femoracetabular Impingement"[Mesh] OR "Femoracetabular Impingement / etiology"[Mesh] OR "Femoracetabular Impingement / physiopathology"[Mesh] OR Cam impingement OR FAI OR Femoroacetabular impingement OR hip OR Hip injury OR labral tear

**AND**

"Follow-Up Studies"[Mesh] OR 10-year follow-up OR long-term follow-up

**AND**

"Acetabulum / surgery"[Mesh] OR "Arthroscopy"[Mesh] OR "Arthroscopy / methods"[Mesh] OR "Athletic Injuries / surgery"[Mesh] OR "Debridement"[Mesh] OR "Debridement / methods"[Mesh] OR "Femoracetabular Impingement / surgery"[Mesh] OR "Hip Injuries / surgery"[Mesh] OR "Hip Joint / surgery"[Mesh] OR "Joint Capsule / surgery"[Mesh] OR "Joint Diseases / surgery"[Mesh] OR Arthroscopy OR hip arthroscopic surgery OR Hip arthroscopy OR labral repair

**AND**

"Patient Reported Outcome Measures"[Mesh] OR "Patient Satisfaction"[Mesh] OR "Treatment Outcome"[Mesh] OR outcome OR Treatment outcome

**SUMMARY**

("Acetabulum / injuries"[Mesh] OR "Athletic Injuries / physiopathology"[Mesh] OR "Femoracetabular Impingement"[Mesh] OR "Femoracetabular Impingement / etiology"[Mesh] OR "Femoracetabular Impingement / physiopathology"[Mesh] OR Cam impingement OR FAI OR Femoroacetabular impingement OR hip OR Hip injury OR labral tear) AND ("Follow-Up Studies"[Mesh] OR 10-year follow-up OR long-term follow-up) AND ("Acetabulum / surgery"[Mesh] OR "Arthroscopy"[Mesh] OR "Arthroscopy / methods"[Mesh] OR "Athletic Injuries / surgery"[Mesh] OR "Debridement"[Mesh] OR "Debridement / methods"[Mesh] OR "Femoracetabular Impingement / surgery"[Mesh] OR "Hip Injuries / surgery"[Mesh] OR "Hip Joint / surgery"[Mesh] OR "Joint Capsule / surgery"[Mesh] OR "Joint Diseases / surgery"[Mesh] OR Arthroscopy OR hip arthroscopic surgery OR Hip arthroscopy OR labral repair) AND ("Patient Reported Outcome Measures"[Mesh] OR "Patient Satisfaction"[Mesh] OR "Treatment Outcome"[Mesh] OR outcome OR Treatment outcome)
